# Hypertrophic cardiomyopathy caused by a heterozygous variant in TTR gene: A case report

**DOI:** 10.1097/MD.0000000000033752

**Published:** 2023-05-17

**Authors:** Huayuan Yuan, Ya Lin, Jiao Wang, Jialian Li, Xuefeng Chen, Yulong Guo, Jiong Tang

**Affiliations:** a Department of Cardiology, Fuwai Yunnan Cardiovascular Hospital, Kunming, Yunnan Province, China.

**Keywords:** hypertrophic cardiomyopathy, transthyretin, TTR

## Abstract

We report a rare case of hypertrophic cardiomyopathy (HCM) caused by a heterozygous variant in TTR gene.

**Patient concerns:**

The proband had been vomiting without obvious inducement since the age of 27, accompanied by the expulsion of stomach contents. At the age of 28, she began to suddenly syncope.

**Diagnosis:**

Cardiac magnetic resonance showed thickening of the right ventricular lateral wall and ventricular septum. The left ventricular diastolic function was limited. Targeted Sanger sequencing validates the presence of mutation p.Leu75Pro in TTR gene.

**Interventions and outcomes:**

After admission to hospital for syncope, she was given metoprolol tablets 25 mg bid, spironolactone tablets 20 mg qd, and trimetazidine 20 mg tid. Her symptoms improved after taking the medicine.

**Lessons:**

The results of this case show that HCM caused by TTR mutation is not easy to be identified and treatment is easy to be delayed. Therefore, high-risk patients with amyloidosis should be evaluated as soon as possible. Timely diagnosis of HCM caused by TTR mutation before irreversible organ damage is essential for proper treatment and better outcomes.

## 1. Introduction

Hypertrophic cardiomyopathy (HCM) is a cardiovascular disease with various clinical manifestations and natural courses. The incidence of HCM in adults is 0.2 %, which is the main cause of sudden cardiac death in young people (<35 years old).^[1]^ Although the etiology of sudden cardiac death is not clear, genetic factors have been shown to play a role, presenting an autosomal dominant as the major pattern of inheritance. Clear mutations are found in 50 to 60% of the patients with HCM, and the most common pathogenic genes are MYH7 and MYBPC3.^[2]^ In contrast, the number of patients with p.Leu75Pro variant in TTR gene is limited.

## 2. Case presentation

Herein, we report a unique case of HCM in a 28-year-old female who presented with left ventricular diastolic dysfunction, and significant thickening of both the left ventricular wall and the anterior wall of the right ventricle. Simultaneous sinus rhythm, occasional premature ventricular contractions, and occasional premature atrial contractions were also detected. Twenty-four-hour SDNN value decreased moderately.

By detecting 261 genes related to HCM, dilated cardiomyopathy, and other hereditary cardiomyopathies or channelopathies, the heterozygous variation of the TTR gene [NM_000371] was revealed. The TTR gene encodes transthyroxine protein, and this mutation causes reduced stability of transthyroxine tetramer and increased amyloid accumulation, leading to HCM. This report focuses on the gene detection results and clinical examination results of this patient.

### 2.1. History of presentation

The proband was a 28-year-old female of non-consanguineous Asian parents. She had experienced recurrent vomiting without obvious inducement during the past year, accompanied by the expulsion of stomach contents. She repeatedly went to the local hospital for examinations but failed to find out the cause. The patient began to have sudden syncope several months ago. After examinations in our hospital, she was finally diagnosed as HCM by echocardiography and cardiac MRI.

**Figure 1.**
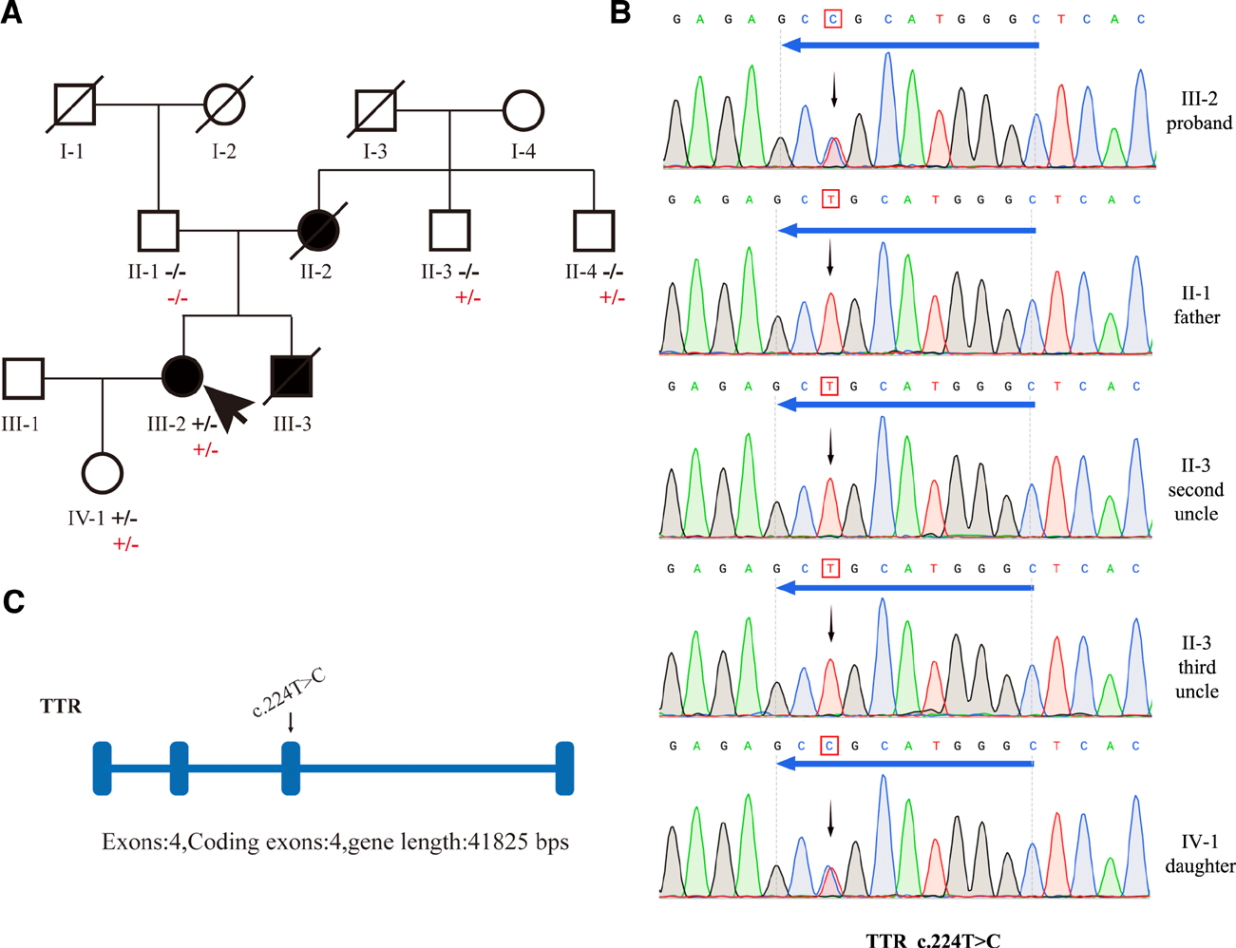
Pedigree and mutation analysis of the family. (A) Pedigree analysis of the proband’s family. The arrow points out the proband (III-2) with the heterozygous mutation of the TTR gene:NM_000371.4 (TTR):c.224T > C (p.Leu75Pro). Circles and squares correspond to female and male individuals, respectively. Affected family members are denoted in black. The mutation was indicated −/− if negative and +/− if heterozygous. Among them, the TTR gene is represented by black words, and the TMPO gene is represented by red words. (B) Pyrogram profiles for variant verification by Sanger sequencing, indicating the sequenced results in III-2 (c.224T > C mutant type). The arrows show the mutation position NM_000371: c.224T > C in the TTR gene. (C) The mutation is located in the third exon of the TTR gene.

Both her mother (II-2) and younger brother (III-3) died suddenly of HCM, one in her 30s and the other in his 20s. Both suffered recurrent vomiting when they were alive, the same symptoms as the patient herself. Her second and third uncles (II-3; II-4) reported themselves as healthy. Her father's (II-1) and daughter’s (IV-1) health status was unknown. A 4-generation family pedigree is shown in Figure 1A.

### 2.2. Investigations

#### 2.2.1. Clinical workup.

Echocardiography revealed a thickening of the left and right ventricular walls. Cardiac MRI showed that the right ventricular lateral wall and the ventricular septum thickened, and the left ventricular diastolic function was limited. These symptoms may suggest HCM. ECG test showed sinus rhythm, occasional ventricular premature beat, and occasional atrial premature beat. Twenty-four-hour SDNN value decreased moderately, according to heart rate variability analysis.

#### 2.2.2. Genetic workup.

HCM may be caused by mutations in multiple genes associated with genetic cardiomyopathy or channelopathies. To identify the potential genetic defects responsible for the observed cardiac phenotype, a commercial panel of the Personal Genome Machine (from Thermo Fisher Scientific) was used to perform targeted sequence analysis of 216 genes related to hereditary cardiomyopathy or channelopathies, including AARS2, CACNA2D1, PSEN2, TTR, TMPO, etc. The heterozygous variant c.274G > T; p.Gly92Cys (rs1201669937) in the TMPO gene (NM_003276) was detected by targeted sequencing of the proband and her father, second uncle, third uncle, and daughter (Table 1). Although both the uncles carried the same variant in the TMPO gene as the proband, neither of them was afflicted with the disease (Fig. 1A). We thus believe that TMPO gene mutations may not be the cause of cardiomyopathy due to the absence of segregation in the family. In addition, targeted sequencing also detected the heterozygous variant c.224T > C; p.Leu75Pro (rs121918079) in the TTR gene (NM _ 000371) (Table 1). Sanger sequencing validates the presence of mutation p.Leu75Pro in TTR gene (Fig. 1B).

**Table 1:** Deleterious variants in TTR gene detected in Sanger sequencing.

Gene	Transcript	Genomic position	DNA change	Protein change	Zygosity	rs id
TMPO	NM_003276	chr12:98909919	c.274G > T	p.Gly92Cys	Heterozygous	rs1201669937
TTR	NM_000371	chr18:29175106	c.224T > C	p.Leu75Pro	Heterozygous	rs121918079

The heterozygous variant c.224T > C is located in exon 3 of the TTR gene (Fig. 1C) and results in the mutation of the 75th amino acid of the encoded protein from leucine to proline. The substitution of leucine with proline is predicted to be deleterious by 3 in silico prediction algorithms (SIFT, PolyPhen2, MutationTaster). The population frequency of this mutation site is not recorded in gnomAD and ExAC databases. Being trans with a known pathogenic variant in a known autosomal dominant disease that fits the phenotype of the patient well, the variant is classified as pathogenic according to published standards and guidelines by the American College of Medical Genetics and Genomics. This heterozygous genotype is considered diagnostic.

### 2.3. Treatment

After admission to the local hospital for sudden syncope for the first time, the patient was treated with metoprolol and exhibited reduced vomiting afterward. In the same year after another syncope, she went to our hospital for treatment. Metoprolol tablets 25 mg bid, spironolactone tablets 20 mg qd, and trimetazidine 20 mg tid were prescribed. Her symptoms improved with no vomiting or syncope observed.

## 3. Discussion

In this report, we describe HCM in a 28-year-old proband carrying the heterozygous variant c. 224T > C in TTR, which is located in the third exon of the TTR gene. We also present the clinical examination results and genetic findings of this patient.

Pathogenic variants in the TTR gene are known to be associated with hereditary transthyretin amyloidosis, which is inherited in an autosomal dominant manner. Amyloidosis is a clinical syndrome caused by the deposition of amyloid substances between cells of various organs in the body for various reasons, which leads to gradual functional failures of involved organs. Its clinical manifestations mainly include amyloid polyneuropathy and cardiomyopathy, but amyloidosis vitreous opacity, amyloidosis of meningeal, and amyloidosis of cerebral vessels may occur as well.

TTR encodes transthyretin, one of the 3 types of prealbumin. Transthyretin is mainly synthesized in the liver and brain choroid plexus and secreted into the blood and cerebrospinal fluid. It transports thyroid hormones in plasma and the cerebrospinal fluid, and also participates in the plasma transport of retinol (vitamin A) through complexation with retinol-binding proteins.^[3]^ TTR also plays a role in other intracellular processes including proteolysis, nerve regeneration, autophagy, and glucose homeostasis. It is a highly stable tetrameric protein. But this conformational stability decreased due to a change in the charge of the monomer caused by a mutation in the gene (c. 224T > C).^[4]^ In the process, the tetramer is decomposed and degraded into monomers,^[5]^ which produce a variety of amyloid fibers, causing abnormal aggregation of amyloid fibers in various organs of the body, such as peripheral nerves or cardiac cells, leading to amyloid polyneuropathy and cardiomyopathy. We speculate that the p.Leu75Pro mutation in TTR is the cause of the above changes and finally leads to the development of HCM in patients.

Through the verification of the patient and her relatives, the patient carries a heterozygous variant of the TTR c.224T > C locus. Her mother died of HCM. However, neither the father with unknown health status nor the uncles who self-reported healthy carried the variation (Fig. 1A and B).

The most common pathogenic TTR mutations are Val22Ile and Val30Met.^[6]^ The Val22Ile variant occurs in 8% of suspected cardiac amyloidosis in Black/African American patients, while the Val30Met variant is prevalent in Japan, Sweden, and Brazil.^[7]^ In contrast, the number of patients with p.Leu75Pro variant is limited. Previously, the p.Leu75Pro variant found in our proband was detected in twelve patients with amyloidosis and published in 5 reports.^[8–12]^ This variant is transmitted in an autosomal dominant manner, resulting in hereditary transthyretin amyloidosis. The disease causes amyloid deposits in various organs of the body and shows heterogeneous phenotypes. Patients can be characterized by multiple neuropathy,^[8–10,12]^ cardiomyopathy,^[8,9]^ gastrointestinal symptoms,^[8–11]^ impaired renal function,^[11]^ vitreous opacity,^[9,10]^ etc. Seven transthyretin amyloidosis patients in one family carried the variation, and at least 2 healthy family members did not, which was consistent with the co-segregation of variation and disease.^[10]^ A number of in vitro experiments showed that the p. Leu75Pro mutation could significantly reduce the stability of transthyretin tetramer and increase amyloidosis.^[13–16]^ In addition, amyloid fiber deposition was found in the intestinal and skin organs of transgenic mice in in vivo experiments.^[16]^ According to the results of single-cell RNA-seq, TTR was slightly expressed in cardiocytes (https://www.proteinatlas.org/).

This disease is not easy to recognize, and when diagnosed, it often progresses to the more advanced stages and is difficult to treat. Early recognition of amyloidosis is needed. Timely diagnosis of HCM resulting from TTR variants before irreversible organ damage is crucial for appropriate treatment and better outcomes.

## Acknowledgments

The index patient is gratefully acknowledged for her generous support to this case report.

## Author contributions

**Conceptualization:** Huayuan Yuan, Yulong Guo, Jiong Tang.

**Data curation:** Huayuan Yuan, Ya Lin.

**Formal analysis:** Huayuan Yuan, Jiao Wang.

**Investigation:** Huayuan Yuan, Jialian Li, Xuefeng Chen.

**Methodology:** Huayuan Yuan, Yulong Guo, Jiong Tang.

**Writing – original draft:** Huayuan Yuan, Ya Lin, Jiong Tang.

**Writing – review & editing:** Yulong Guo, Jiong Tang.
